# Malignant Transformation of Testicular Teratoma to PNET, Adenocarcinoma, and Osteosarcoma with Complete Remission after Surgery and Combination Chemotherapy in a Young Adult Male

**DOI:** 10.1155/2018/8460603

**Published:** 2018-10-03

**Authors:** Angela Shaw, Miriam Morrell, Annikka Weissferdt, Andrea Hayes-Jordan, Douglas Harrison

**Affiliations:** ^1^Department of Pediatrics, The University of Texas MD Anderson Cancer Center, Houston, TX, USA; ^2^Department of Pathology, The University of Texas MD Anderson Cancer Center, Houston, TX, USA; ^3^Department of Surgical Oncology, The University of Texas MD Anderson Cancer Center, Houston, TX, USA

## Abstract

Mixed germ cell tumors (GCT) with teratoma components can transform into somatic malignancies which can include histologies outside of traditional germ cell lineages. We describe a case of an 18-year-old man with a metastatic testicular GCT with both mature and immature teratoma components containing malignant transformation into multiple histologies including PNET in the primary testicular tumor and osteosarcoma in a separate pulmonary metastatic lesion. Management with targeted chemotherapy resulted in a durable remission. This is the first reported case that we know of a patient with primary PNET malignant transformation with subsequent metastatic transformation to osteosarcoma.

## 1. Introduction

Germ cell tumors (GCT) of the testis are the most common solid tumors diagnosed in males between the ages of 15 and 40 years [1]. GCTs are typically divided into two major subtypes—seminomatous and nonseminomatous GCT. Nonseminomatous GCT subtype includes embryonal carcinoma, yolk sac tumor, choriocarcinoma, and teratoma. Initial management combines surgical exploration with orchiectomy and diagnostic evaluation of serum tumor markers followed by clinical staging and risk stratification according to the International Germ Cell Cancer Collaborative Group classification system. Depending on risk status, patients may receive systemic chemotherapy which typically incorporates bleomycin, etoposide, and cisplatin (BEP). Prognosis is excellent with a 5-year relative survival rate for males in late adolescence and early adulthood of approximately 96% [2].

GCTs with malignant transformation into somatic histologies are rare, occurring in only 2.7–8.6% of all GCT cases [3]. Malignant transformations from GCT typically arise from mediastinal nonseminomatous GCTs rather than gonadal primary tumors [4]. The development of malignant transformation with sarcomatous components in GCTs tends to occur in nonseminomatous GCTs with teratoma histologies [5]. Primary malignant transformation to sarcoma is the most frequent histology [5, 6], with rhabdomyosarcoma being the most common subtype [4, 6]. Osteosarcoma subtype is rare and has been reported only once [6]. Following sarcoma, transformation to adenocarcinoma and primitive neuroectodermal tumor (PNET) are the next most common histologies [6]. Unlike teratoma, PNET transformation allows for metastatic potential [7] which may render tumors unresectable and difficult to treat.

## 2. Case Report

An 18-year-old man with a past medical history significant for an osteochondroma of the right forearm presented with a three-month history of a slowly enlarging right testicle. At the time of his initial presentation, the testicle had reached 9–10 cm in diameter. The patient was asymptomatic aside from the enlarged testicle without any constitutional symptoms. Tumor markers, including alpha fetoprotein and beta human chorionic gonadotropin, were negative. Family history was significant for a maternal grandmother with uterine and colon cancer both diagnosed in her 60s, a paternal grandfather diagnosed with prostate cancer in his 60s, and a maternal great grandfather diagnosed with leukemia in his 30s.

A unilateral orchiectomy was performed. Pathology was consistent with mature teratoma (90%) and PNET (10%). Chest and abdominal CT scan revealed multiple retroperitoneal lymph nodes matted into a mass. Retroperitoneal lymph node dissection (RPLND) was performed revealing mature and immature teratoma with 60–70% transformation to PNET. This positive lymph node spread prompted second opinion review of the pathology. Second opinion pathologic evaluation of the primary tumor revealed nonseminomatous mixed germ cell tumor composed of mature and immature teratoma (95%, of which 30–50% was PNET), yolk sac tumor (3%), and embryonal carcinoma (2%). Due to the PNET component, ^18^F-FDG-PET (PET) imaging was obtained which revealed a 1 cm PET avid left lower lobe lung nodule with a standard uptake value (SUV) of 2.8. A multidisciplinary team discussed treatment and planned for surgical resection of the lung nodule following initiation of therapy. There was concern that the patient was progressing when the FDG-PET avid nodule was discovered, and there had been considerable delay in confirming the diagnosis. As such, it was decided to proceed with chemotherapy and explore the pathology of the nodule if the patient did not experience adequate treatment response. Given the unusual nature of his case and the presence of several family members with a history of cancer, the patient was offered TP53 genetic testing but declined.

Combination chemotherapy was initiated with an alternating regimen that included cycles of vincristine/doxorubicin/cyclophosphamide and ifosfamide/etoposide given in compressed two-week cycles. The patient tolerated chemotherapy well without any major toxicity aside from intermittent mild myelosuppression.

**Figure 1:**
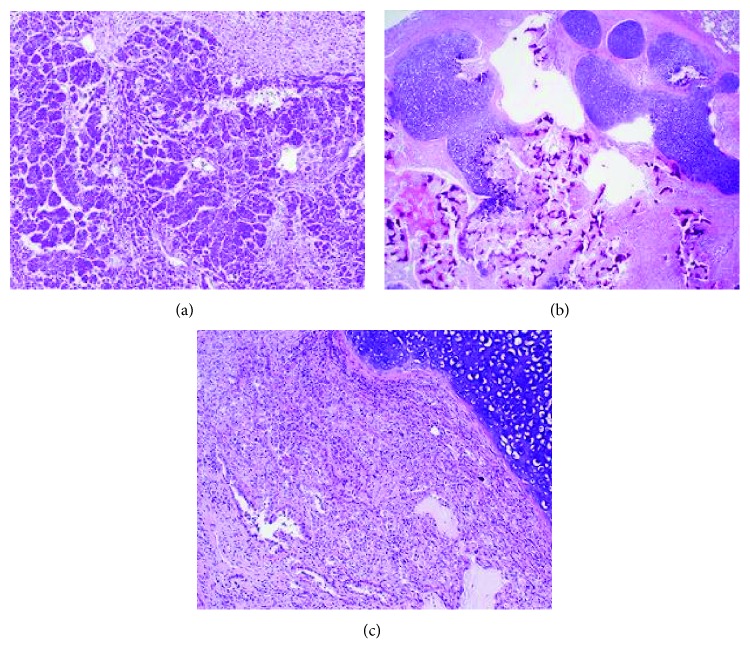
(a) Primitive neuroectodermal tumor component present in the testicular primary tumor and precaval mass. (b) Metastatic teratoma to the lung with transition to areas of osteosarcoma. (c) Metastatic teratoma to the lung with adjacent adenocarcinoma component.

Reevaluation of the PET avid lung nodule occurred prior to cycle 5 and showed persistent avidity with an SUV of 2.92. A wedge resection of the left lower lung lobe nodule was completed following cycle 9 of chemotherapy. Pathology of this nodule revealed mature teratoma (40%), osteosarcoma (30%), and adenocarcinoma (30%) components (Figure 1).

Treatment was subsequently amended to an osteosarcoma-based treatment regimen that contained four cycles of cisplatin with doxorubicin again alternating with 3 cycles of ifosfamide and etoposide. Total cumulative dose included doxorubicin 600 mg/m^2^, cisplatin 360 mg/m^2^, ifosfamide 54 g/m^2^, etoposide 3 g/m^2^, and cyclophosphamide 6 g/m^2^. He received dexrazoxane for cardioprotection prior to each doxorubicin infusion for a total cumulative dose of 6 g/m^2^. The patient's end of therapy evaluation was negative for any evidence of disease, and he is alive with no evidence of disease after 18 months.

## 3. Discussion

While germ cell tumors are a common malignancy in young men, it is rare for them to develop somatic malignant transformation. Malignant transformation more commonly arises in mediastinal nonseminomatous germ cell tumors than in gonadal germ cell tumors [4]. Sarcomas, typically rhabdomyosarcoma subtype, and PNET are among the most commonly reported histologic transformations [3–5, 7]. To our knowledge, primary malignant transformation to osteosarcoma has been reported only once [6]. Transformation to multiple distinct solid tumor histologies in the primary tumor has also been previously reported [6, 8]. Prevalence of transformation to multiple distinct histologies in metastases is unknown, with only two reports that included mature and immature teratoma, embryonal rhabdomyosarcoma, and non-Hodgkin's lymphoma components [6] and mature teratoma, choriocarcinoma, and embryonal carcinoma, respectively [8].

Because of the rarity of these tumors, prognostic factors and optimal management of germ cell tumors with somatic malignant transformation are poorly defined. Cisplatin-based chemotherapy regimens have good outcomes in nontransformed malignant germ cell tumors, with a cure rate of greater than 80% [5, 9]. Unfortunately, these regimens have proven to be ineffective in the treatment of malignantly transformed germ cell tumors [10]. In particular, germ cell tumors with PNET transformation are usually resistant to cisplatin-based chemotherapy regimens [7, 11, 12]. Surgical resection remains the mainstay of treatment for germ cell tumors with malignant transformation. However, systemic chemotherapy may be effective when the choice of treatment regimen is directed towards the transformed histology [4] and can improve surgical resectability. Germ cell tumors with somatic transformation into PNET have in certain cases been successfully treated with chemotherapy that includes cycles of vincristine, doxorubicin, and cyclophosphamide alternating with ifosfamide and etoposide. In one study, this resulted in a median survival of 32.7 months [13].

Further research is needed to adequately define treatment paradigms for patients with germ cell tumors transformed into multiple somatic histologies. It has been suggested that treatment for patients with unresectable malignant transformations be tailored towards the specific transformed histology [6]. We have demonstrated that thorough surgical resection and combination chemotherapy targeted towards the most aggressive found histology can lead to disease control and durable remission in patients with gonadal germ cell tumors containing transformation to multiple malignant somatic histologies.
